# Parenting stress and competence in borderline personality disorder is associated with mental health, trauma history, attachment and reflective capacity

**DOI:** 10.1186/s40479-020-00124-8

**Published:** 2020-05-11

**Authors:** Kayla R. Steele, Michelle L. Townsend, Brin F. S. Grenyer

**Affiliations:** School of Psychology, Illawarra Health and Medical Research Institute, University of Wollongong, Wollongong, New South Wales Australia

**Keywords:** Personality disorder, Borderline personality disorder, Parenting, Stress, Competence, Mentalizing, Trauma, Attachment

## Abstract

**Background:**

Individuals with borderline personality disorder (BPD) may experience additional challenges in their parenting role, including increased stress and lower self-efficacy and satisfaction. These difficulties have been shown to impact their children, and may be implicated in the potential intergenerational transmission of personality vulnerabilities.

**Methods:**

Parental stress and competence variables were examined in a cross-sectional study of 284 parents (94.72% female, *M* = 37.37, *SD* = 8.04 years), of which 69 (24.30%) met caseness for BPD criteria. We completed a multivariate analysis of variance to test how parents with ‘high BPD features’ (meeting caseness for BPD) compared to those with ‘low BPD features’ on a range of parenting and mental health variables. Multivariate linear regression modelling was then utilised to explore whether these parenting variables were associated with personality and psychological wellbeing, recalled trauma history, orientation to attachment relationships and reflective capacity.

**Results:**

Individuals high in BPD features experienced more stress and lower competence in their parenting role than those low in BPD features. These parents also reported more personality vulnerabilities, poorer psychological wellbeing, recalled more traumatic experiences in their childhood, were more likely to endorse insecure attachment styles and had poorer reflective capacity. In the regression model, parenting stress and competence was associated with personality traits, general psychological wellbeing, recalled trauma history, attachment style and reflective capacity variables. Parental reflective capacity had the strongest association with parenting stress, satisfaction, efficacy, the perception of having a difficult child and a difficult parent-child relationship, and psychological wellbeing had the greatest association with parenting distress.

**Conclusions:**

Parents who were able to imaginatively enter the subjective world of the child and hold the child’s mind in mind with less certainty, reported reduced parenting stress and greater parenting satisfaction and efficacy. Helping to improve personality and mental health functioning, increasing parental reflective capacity and strengthening parent-child attachment relationships, may reduce parenting stress and increase parenting competence in individuals with BPD.

## Background

Personality disorder is a complex and severe mental health issue that is characterised by pervasive and enduring difficulties in intra-personal (e.g. identity, self-worth, accuracy of self-view, self-direction), and inter-personal functioning (e.g., ability to develop and maintain close and mutually satisfying relationships, ability to understand others’ perspectives and to manage conflict in relationships) [1]. These difficulties deviate markedly from the expectations of the individual’s culture and are associated with significant distress or impairment in personal, family, social, educational, occupational or other important spheres [1, 2]. Globally, personality disorder affects 7.8% of the general population [3] and as such, is considered a mental health priority area [4].

Borderline Personality Disorder (BPD), characterised by marked distress caused by intense affectivity and impulsivity, interpersonal difficulties and distorted cognitions [2], is common in primary care and mental health settings. Up to 23% of outpatients and 43% of inpatients in Australian mental health services meet criteria for BPD [5], compared to approximately 0.7–2% of the general global population [6]. Although BPD is thought to occur equally amongst men and women in the general global population [7] women are disproportionately represented in clinical settings, comprising of up to 75% of those given a BPD diagnosis in the United States of America according to the Diagnostic and Statistical Manual of Mental Disorders, fourth edition, text revision (DSM-IV-TR) [8]. In the Australian context, recent data from a representative health service found that men and women had similar rates of presentation with a diagnosed personality disorder [9]. However, women were more likely to be referred to a personality disorder service or be offered specialist long-term psychological therapy [9, 10].

For individuals with BPD, emotional dysregulation, high levels of impulsivity (often leading to self-harm and suicidality), and disturbed interpersonal functioning, are thought to lead to difficulties in forming and maintaining inter- and intra-personal relationships [2]. In combination, genetic vulnerability to BPD and negative early experiences with parents and caregivers are considered to put a child at increased risk of developing BPD or experiencing its related features in adulthood [11, 12]. As such, the parent-child relationship is considered an important context for the aetiology and the potential intergenerational transmission of BPD [13].

### Parenting challenges associated with borderline personality disorder

Parents with BPD appear to experience additional emotional and behavioural challenges in their parenting role. For these individuals, fluctuations in mental wellbeing, difficulties with expressing appropriate empathic responses, maintaining a stable and safe environment, parent-child role confusion (e.g. parentification of children) and interpersonal conflict appear to exacerbate the everyday challenges of being a parent [14]. Mothers with BPD are considered to be particularly at risk, with this group more likely to demonstrate misattuned speech and behaviour with their infants, including critical, intrusive and frightened or frightening comments and behaviours, and increased role confusion compared to those with depression and healthy controls [15, 16]. Additionally, these parents also differ in their perceptions of their parenting ability. Self-report measures assessing parenting perceptions show that mothers with BPD report less competence (satisfaction and efficacy) and more distress in their parenting role, and it has been hypothesised that these difficulties may inadvertently contribute to a greater likelihood of mothers with BPD struggling with safe parenting of their children [17]. Thus, a mother’s level of distress and sense of competency may profoundly affect parenting capacity and compromise responsiveness and sensitivity to the child.

Children of parents who struggle with personality difficulties, particularly BPD, demonstrate lower Apgar scores, and greater rates of prematurity and special care nursery referral during early infancy compared to controls [18]. Whilst at 12-months of age, infants of mothers with BPD have been found to show lower levels of “availability for positive engagement”, and greater attachment disorganization compared to controls [19]. Young children aged four to seven whose mothers have BPD tell stories that include more parent-child role reversal, fear of abandonment, negative parent-child relationship expectations, incongruent and shameful representations of self and poorer emotion regulation, compared to controls [20]. During adolescence, children aged 11 to 18 years (averaging 15.52 years) of mothers with BPD, have been found to exhibit significantly more emotional (e.g. anxiety/depression, self-esteem and emotional problems more broadly), attentional and behavioural problems (e.g. delinquency, aggression and behavioural problems more broadly), and more suicidal tendencies, than children of mothers with depression only, children of mothers with no psychiatric condition, or children of mothers with ‘cluster C’ personality disorders [14]. Given the range of challenges encountered by parents with personality disorder and the possible impact on their children across various developmental stages, identifying key mechanisms may help to prevent parents inadvertently transferring these difficulties onto the child. There is consequently a great need to further understand and modify the putative intergenerational transmission of personality vulnerabilities.

### Potential mechanisms underlying parenting challenges

There are a number of mechanisms that have been hypothesised to underly parenting challenges for individuals with BPD. However, early childhood trauma, attachment and reflectivity capacity or ‘mentalizaton’ have historically received attention in the literature exploring parenting and BPD [13]. Many individuals with BPD (up to 84% in some studies) [21] retrospectively describe experiences of bi-parental neglect and emotional abuse before the age of 18. This history of early trauma may be associated with challenges for individuals with BPD in their own parenting role, particularly for those who find the experience triggers painful memories of early abuse or neglect that they may attempt to overcompensate with “overprotection” [14]. A parent’s history of early trauma may also be associated with negative outcomes for their children. For example, in a recent study [22] of youth brought to the attention of child protective services for history of maltreatment, substance abuse and family violence, 34.3% of mothers had a previous diagnosis or met criteria for BPD. Half of these mothers had also experienced childhood maltreatment and were also investigated by child protective services. Given the elevated rates of childhood abuse and neglect reported by individuals with BPD and the potential for the parent-child relationship to trigger traumatic memories from the parent’s family of origin, it seems plausible that a parent’s recalled trauma history may be associated with their feelings of stress and competence in their parenting role in the present. However, further investigation is needed to support this proposition.

A secure attachment relationship in early childhood is thought to lay the foundation for a child’s capacity to develop secure relationships in adolescence and adulthood, the capacity for emotional regulation and reflectivity capacity. Conversely, if children do not have their attachment needs met or receive inappropriate responses from their caregiver (such as lack of response, inconsistent or abusive responses), they may develop maladaptive internal working models that result in insecure attachment [23]. Individuals with BPD have been found to more frequently endorse insecure attachment styles, particularly preoccupied, fearful or unresolved, than the general population [24]. Moreover, mothers with BPD are more likely to have infants who also exhibit insecure or disorganised attachment styles, with intrusive or insensitive maternal relatedness thought to be a key factor impacting infant attachment organisation [19]. Based on previous research, the manner or quality of a parent’s way of relating to their child may also be influenced by their psychological wellbeing (including their personality difficulties and perceived parenting stress) [16, 17] and their relationship to their self (including their role as a parent and parent-child role confusion) [14]. However, the relationship between attachment and perceptions of parenting stress and competence for individuals with BPD is not yet known. Further investigation may assist us to identify possible mechanisms underlying parentings challenges faced by individuals with BPD.

Impairments in reflective capacity have been implicated in various psychiatric disorders, including BPD [25–30]. Mentalization or ‘reflective functioning’ is the process through which a person is able to make meaning of their own behaviour and infer the mental states of others (i.e., thoughts, feelings and beliefs) and has been described as the individual’s ability to ‘hold others’ minds in mind’ [31–33]. In the context of the parent-child relationship, parental reflective capacity or ‘parental reflective functioning’ describes a parent’s capacity to reflect upon their own child’s internal mental experience and to understand behaviour in the light of the child’s underlying mental states and intentions, and in doing so ‘hold the child’s mind in mind’ [34, 35]. Parental reflective capacity has been found to be related to mother-infant attachment [36, 37] and sensitive and responsive caregiving [38, 39]. It also seems plausible that a parent’s ability to enter and reflect upon the subjective world of their child may also impact their levels of stress and their feelings of competence in parenting role. However, there is currently a gap in literature in regard to the putative relationship between parenting reflective capacity and perceptions of parenting stress and competence. Through exploring this relationship, we hope to come closer to identifying key mechanisms underlying a small subset of the difficulties that many individuals with BPD face in their parenting role.

### The current study

The current study examines how a parent’s subjective rating of their stress and competence may be associated with difficulties in the parent’s life, including their personality and other mental health challenges, their attachment, reflective capacity, and also recalled experiences from their early family environment. In concordance with previous literature demonstrating the challenges faced by individuals with BPD, we hypothesise that:

1. Individuals higher in BPD features will report greater parenting stress and lesser parenting competence compared to those lower in BPD features.
2. Those higher in BPD features are also hypothesised to report additional challenges, including lesser psychological wellbeing, lesser parental reflective capacity, greater attachment difficulties, and will be more likely to report trauma in their early family environment, compared to those lower in BPD features.
3. Taken together, personality difficulties, psychological wellbeing, trauma history, attachment and parental reflective capacity are hypothesised to predict parenting stress and competence for the entire sample.

## Methods

### Participants and procedure

Participants responded to a call for volunteers to complete an online survey on “Being a parent today: A study of stress and satisfaction” via online forums related to parenting, personality disorder, and general mental health in 2018. To be eligible for inclusion in this study participants had to be 19 years and over, currently involved in parenting at least one child aged zero to 19 years, and complete all psychometric measures. Participants were invited to complete standard psychometric measures of parenting stress and competence, personality and mental health, attachment, reflective capacity and trauma history on the online survey platform. Participants gave explicit consent to participate after reading the participant information sheet online, and there was no payment made for their contribution. Data were analysed using multivariate analyses of variances and multivariate linear regression modelling. All statistical analyses were conducting using IBM SPSS statistics v22.

The sample comprised 284 participants, the majority being women who were likely to be married, had completed or were undertaking tertiary education, and working full or part-time. The age of participants varied from 22 to 58 years (*M* = 37.37). Whilst the age range of participant’s children ranged from zero (i.e. participants who completed the survey whilst pregnant) to 41 years (*M* = 9.26). In regard to clinical features, the majority of participants (71.13%) had previous or current mental health concerns and had also previously or were currently engaged in treatment (65.85%). Approximately one quarter (24.30%) of the sample endorsed seven or more BPD features and therefore met caseness for BPD according to the MSI-BPD [40]. Participant demographic and clinical features are further described in Table 1*.*

**Table 1:** Frequencies and percentages of sample characteristics (*N* = 284)

Characteristic	*n* (%)
Gender	
Female	269 (94.72)
Male	14 (4.93)
Other	1 (0.35)
Relationship status	
Single (never married)	20 (7.04)
Married	166 (58.45)
Separated	23 (8.10)
Widowed	2 (0.70)
Divorced	12 (4.23)
Living together	50 (17.61)
In a relationship (living apart)	4 (1.41)
Other	6 (2.11)
Education	
High school (not completed)	17 (6.69)
High school (completed)	23 (8.10)
Technical qualification	36 (12.68)
University degree (not completed)	55 (19.37)
Bachelor’s degree	79 (27.82)
Postgraduate degree	65 (22.89)
Other	9 (3.17)
Employment status	
Unemployed	24 (8.45)
Working part-time	86 (30.28)
Working full-time	81 (28.52)
Studying part-time	16 (5.63)
Studying full-time	32 (11.27)
Other	35 (12.32)
Income (yearly or joint yearly)	
Under $15,000	14 (4.93)
$15,000–$29,999	32 (11.27)
$30,000–$49,999	29 (10.21)
$50,000–$74,999	40 (14.08)
$75,000–$99,999	49 (17.25)
$100,000–$150,000	72 (25.35)
Over $150,000	47 (16.55)
Mental health concerns (previous or current)	
Yes	202 (71.13)
No	82 (28.87)
Treatment seeking (previous or current)	
Yes	187 (65.85)
No	97 (34.15)
Meets caseness for BPD	
Yes	69 (24.30)
No	215 (75.70)
Child’s gender:	
Female	278 (50.92)
Male	268 (49.08)

### Measures

#### Parenting stress

Parenting stress was assessed using the Parenting Stress Index 4th Edition – Short Form (PSI-4-SF) [41], an abbreviated version of the full-length Parenting Stress Index 4th Edition (PSI-4). The PSI-4-SF is measure of the overarching domains of parenting stress in which participants are asked to respond to 36-items using a 5-point Likert scale ranging from ‘strongly agree’ to ‘strongly disagree’. The PSI-4-SF is delineated into three subscales, including: parental distress, parent-child difficult interaction, and difficult child, with these three domains combined to form a total stress scale. Scores obtained within the 16th to 84th percentile are considered normal, whilst scores in the 85th to 89th percentile are consider high and those in the 90th percentile or higher are considered clinically significant. The PSI-4-SF has been found to show good internal consistency for its subscales, ranging from .88 (difficult child) to .95 (total stress), test-retest reliability and content and construct validity [41]. In the present analysis, total stress scale, parenting distress and parent-child dysfunctional interaction were of primary interest. Cronbach’s alpha estimates for the three subscales of the PSI-4-SF showed excellent reliability for parent-child difficult interaction (*α* = .91) and total stress score (*α* = .95), and good reliability for parental distress (*α* = .88), and difficult child (*α* = .89).

#### Parenting competence

Parenting competence was assessed using the Parenting Sense of Competence Scale (PSOC) [42]. The PSOC is a 17-item self-report measure with each item rated on a 6-point Likert scale ranging from 1 ‘strongly disagree’ to 6 ‘strongly agree’. The PSOC is further differentiated into two subscales: parenting efficacy and parenting satisfaction, with higher scores indicating increased efficacy and satisfaction. The PSOC has been reported as having adequate reliability and validity in the literature [42, 43]. In the present analysis, Cronbach’s alpha estimates also showed good reliability for parental satisfaction (*α* = .82) and acceptable reliability for parental efficacy (*α* = .77).

#### Personality traits and severity

Personality was assessed dimensionally across several different personality trait domains using the Personality Inventory for the Diagnostic and Statistical Manual of Mental Disorders 5th edition (DSM-5) – Brief Form (PID-5-BF) [2]. The PID-5-BF was developed by the DSM-5 Personality and Personality Disorders workgroup for the assessment of the alternative trait model for DSM-5. It is a 25-item self-report measure with each item rated on a 4-point Likert scale ranging from 0 ‘very false or often false’ to 3 ‘very true or often true’. The PID-5-BF assesses the overall severity of personality disorder, with scores ranging from zero to 75 and higher scores indicating greater overall personality dysfunction. The measure also assesses five personality trait domains including negative affect, detachment, antagonism, disinhibition, and psychoticism, with each trait domain ranging in score from zero to 15 and higher scores indicating greater dysfunction in the specific personality trait domain. The PID-5-BF is a relatively novel measure but has been found to have adequate internal consistency for personal trait domains and validity as a screening measure of dimensional maladaptive personality traits [44]. In our study, Cronbach’s alpha estimate for these five different personality trait domains were considered to demonstrate acceptable reliability for negative affect (*α* = .78), detachment (*α* = .71), disinhibition (*α* = .75) and psychoticism (*α* = .77), and poor reliability for antagonism (*α* = .63). Personality pathology was also assessed categorically using the McLean Screening Instrument for Borderline Personality Disorder (MSI-BPD) [40]. The MSI-BPD is a 10-item binary measure of borderline personality symptomology, with a score of seven or more indicating that a person may meet criteria for a diagnosis of BPD. The MSI-BPD has shown good test-retest reliability [40], concurrent validity [45] and criterion validity [46]. In the current study, Cronbach’s alpha estimate for the MSI-BPD showed good reliability (*α* = .84).

#### Psychological wellbeing

Psychological wellbeing was assessed using the Mental Health Inventory (MHI-5) [47]. The MHI-5 is a 5-item self-report measure that is derived from the mental health dimension of the Medical Outcomes Study 36-Item Short Form Health Survey (SF-36). An example item is “During the past month, how much of the time were you a happy person?” Participants are asked to respond to using a 6-point Likert scale ranging from 1 ‘all of the time’ to 6 ‘none of the time’. The MHI-5 has been found to be valid, and reliable for use with different subgroups and a range of different cultures [48]. In the current study, Cronbach’s alpha estimates for the MHI-5 showed good reliability (*α* = .88).

#### Trauma history

Parent’s own trauma history was assessed using the Childhood Trauma Questionnaire (CTQ-SF) [49]. The CTQ-SF is a 28-item self-report that screens for histories of abuse and neglect on a 5-point Likert scale ranging from 1 ‘never true’ to 5 ‘very often true’. The CTQ-SF enquires about five different types of maltreatment, namely emotional, physical, and sexual abuse, and emotional and physical neglect. The CTQ-SF has been validated with data from 2000 clinical and non-referred participants by its authors [49]. Reliability coefficients for the CTQ-SF range from .92 for sexual abuse to .66 for physical neglect, test-retest intraclass correlations ranged from *r* = .79 for physical neglect to overall *r* = .86. Satisfactory content, construct, and concurrent validity was also reported. In the present study the CTQ-SF also showed excellent reliability for emotional abuse (*α* = .91), physical abuse (*α* = .90), and sexual abuse (*α* = .96), good reliability for physical neglect (*α* = .80), and questionable reliability for emotional neglect (*α* = .62).

#### Attachment style

Adult attachment style was assessed using the Relationship Questionnaire (RQ) [50]. The RQ is a 4-item self-report measure based on Bowlby’s [51] ‘internal working model’ in which participants are asked to respond to four paragraphs representing four attachment styles (secure, dismissing, preoccupied, fearful) using a 7-point Likert scale ranging from 1 ‘disagree strongly’ to 7 ‘agree strongly’. In the current study, attachment is measured dimensionally by style (i.e. secure, dismissing, preoccupied, fearful), rather than categorically (e.g. secure vs. insecure). The RQ is founded on a sound theoretical basis and has good convergence with other attachment measures [52, 53] and has been shown to be predictive of therapy rates of change [54]. However, its reliability is less well studied because the nature of the scale does not lend itself to reliability estimates [55].

#### Reflective capacity

Participants’ parental reflective capacity, or their ability to reflect on the mental states of their child and their role as a parent, was assessed using the Parental Reflective Function Questionnaire (PRFQ) [56]. The PRFQ is a 36-item self-report measure that utilises a 7-point Likert scale ranging from 1 ‘strongly disagree’ to 7 ‘strongly agree’. The PRFQ is comprised of three subscales, namely: non-mentalizing mode, certainty about mental states and interest/curiosity in mental states. The PRFQ is a relatively novel measure, yet preliminary research suggests that it has good reliability and validity [56]. In this study the PRFQ showed acceptable reliability for non-mentalizing mode (*α* = .71), certainty about mental states (*α* = .76) and interest/curiosity about mental states (*α* = .72).

## Results

### Comparing high and low BPD features on parenting and concomitant variables

We divided the sample into a ‘high BPD features’ and ‘low BPD features’ group, with the ‘high BPD features’ group defined as endorsing seven or more BPD features on the MSI-BPD [40] and thus meeting caseness for BPD. Groups were compared on a number of parenting stress and competence, personality, psychological wellbeing, trauma history, attachment and reflectivity capacity variables. Results of these analyses are presented in Table 2*.*

**Table 2:** Comparison of parenting stress and mental health variables between parents with high and low BPD features (*N =* 284)

Variables	*M (SD)*		*F*	*p*	*η*_*p*_^*2*^
	High BPD features (MSI-BPD) *N* = 69	Low BPD features (MSI-BPD) *N* = 215			
PSI-4-SF Parenting stress					
Total Stress	110.46 (2.98)	87.94 (1.64)	45.99	<.001	.14
Distress	42.86 (1.03)	31.30 (.58)	96.20	<.001	.25
Difficult child	35.77 (1.13)	30.55 (.64)	16.27	<.001	.06
Difficult relationship	31.84 (1.19)	26.09 (.67)	17.81	<.001	.06
PSOC Parenting competence					
Satisfaction	28.62 (.70)	34.18 (.40)	47.88	<.001	.15
Efficacy	28.39 (.79)	35.60 (.45)	63.39	<.001	.18
PID-5-BF Personality disorder					
Severity	33.28 (1.05)	15.81 (.60)	208.78	<.001	.43
Negative affect	10.03 (.37)	5.48 (.21)	112.96	<.001	.29
Detachment	7.57 (.32)	3.47 (.18)	122.69	<.001	.30
Antagonism	2.71 (.25)	1.77 (.14)	10.62	<.001	.04
Disinhibition	5.71 (.31)	2.22 (.18)	93.88	<.001	.25
Psychoticism	7.26 (.34)	2.87 (.19)	124.59	.001	.31
MHI-5 Psychological wellbeing	16.22 (.52)	22.29 (.29)	104.04	<.001	.27
CTQ-SF Trauma history					
Emotional abuse	15.75 (.70)	10.74 (.40)	38.68	<.001	.12
Physical abuse	10.58 (.62)	8.44 (.35)	9.14	.003	.03
Sexual abuse	10.29 (.66)	7.37 (.38)	14.69	<.001	.05
Emotional neglect	16.09 (.46)	13.56 (.26)	23.00	<.001	.08
Physical neglect	9.55 (.44)	7.43 (.25)	17.89	<.001	.06
RQ Attachment style					
Secure	2.96 (.21)	4.34 (.12)	32.36	<.001	.10
Fearful	5.36 (.21)	3.61 (.12)	53.31	<.001	.16
Preoccupied	4.26 (.20)	3.00 (.12)	29.30	<.001	.09
Dismissing	4.10 (.21)	4.07 (.12)	.02	.896	.00
PFRQ Reflective capacity					
Non-mentalizing	2.77 (.10)	1.91 (.06)	59.77	<.001	.18
Certainty about mental states	3.41 (.12)	3.78 (.07)	7.36	.007	.03
Interest/curiosity in mental states	5.64 (.09)	5.81 (.05)	2.55	.112	.01

*Notes. PSI-4-SF* Parenting Stress Index 4th Edition – Short Form, *PSOC* Parenting Sense of Competency Scale, *PID-5-BF* Personality Inventory for DSM-5 – Brief Form, *MHI-5* Mental Health Inventory, *CTQ-SF* Childhood Trauma Questionnaire – Short Form, *RQ* Relationship Questionnaire, *PRFQ* Parental Reflective Functioning Questionnaire

Parents with high BPD features reported significantly greater parenting stress, distress, difficult child and difficult parent-child relationships compared to those with low BPD features, with medium to large effect sizes observed. Parents with high BPD features also reported significantly lower satisfaction and efficacy. This group also had significantly greater personality disorder severity, were more likely to endorse the personality trait domains of negative affect, detachment, antagonism, disinhibition, and psychoticism, and had lower psychological wellbeing. They also recalled significantly higher emotional abuse, physical abuse, sexual abuse, emotional neglect and physical neglect in their childhood. Parents with high BPD features were also more likely to endorse a fearful or preoccupied attachment style. Presence of dismissive attachment did not significantly differ between the groups (*p* = .90). Parents with high BPD features also showed poorer reflective capacity (indicated by increased non-mentalizing and being ‘overly certain’ about mental states) than those with low BPD features, with large and small effect sizes. Demonstrating an interest and curiosity about mental states did not differ between the groups (*p* = .11).

### Relationship between parenting and concomitant variables

For the entire sample of 284 participants, bivariate correlations (Pearson’s *r*) showed significant relationships between parenting stress and competency variables and mental health, reflective capacity and most trauma history (with the exception of physical and sexual abuse) and attachment (with the exception of dismissing-avoidant attachment). Scores ranged in magnitude from small (*r* = .12, *p* < .05) to moderate (*r* = .65, *p* < .01). Results of the correlational analyses are presented in Table 3*.*

**Table 3:** Correlational matrix of parenting and mental health variables (*N =* 284)

Variables	1	2	3	4	5	6	7	8	9	10	11	12	13	14	15	16	17	18	19	20	21	22	23	24	25	26	*M (SD)*
1. PSI-4-SF Total Stress	–																										93.41(25.85)
2PSI-4-SF Distress	.81^**^	–																									34.11(9.85)
3. PSI-4-SF Difficult relationship	.90^**^	.54^**^	–																								27.49(10.14)
4. PSI-4-SF Difficult child	.91^**^	.59^**^	.81^**^	–																							31.82(9.60)
5. PSOC Satisfaction	−.62^**^	−.52^**^	−.57^**^	−.53^**^	–																						32.83(6.26)
6. PSOC Efficacy	−.75^**^	−.75^**^	−.58^**^	−.63^**^	.63^**^	–																					33.85(7.22)
7. MHI-5 Psych Wellbeing	−.57^**^	−.68^**^	−.40^**^	−.42^**^	.51^**^	.59^**^	–																				20.82(5.03)
8. PID-5-BF Severity	.52**	.60**	.41**	.36**	.44**	.56**	−.65	–																			20.05(11.51)
9. PID-5-BF Negative affect	.50^**^	.56^**^	.39^**^	.38^**^	−.40^**^	−.54^**^	−.65^**^	.82**	–																		6.58(3.66)
10. PID-5-BF Detachment	.53^**^	.62^**^	.42^**^	.35^**^	−.37^**^	−.48^**^	−.57^**^	.72**	.55^**^	–																	4.46(3.20)
11. PID-5-BF Antagonism	.17^**^	.13^*^	.17^**^	.13^*^	−.18^**^	−.19^**^	−.23^**^	.51**	.26^**^	.14^*^	–																2.00(2.12)
12. PID-5-BF Disinhibition	.26^**^	.32^**^	.19^**^	.16^**^	−.34^**^	−.37^**^	−.35^**^	.75**	.47^**^	.36^**^	.42^**^	–															3.07(3.00)
13. PID-5-BF Psychoticism	.39^**^	.47^**^	.29^**^	.27^**^	−.29^**^	−.42^**^	−.51^**^	.84**	.61^**^	.51^**^	.33^**^	.55^**^	–														3.94(3.41)
14. MSI-BPD Features	.45^**^	.56^**^	.31^**^	.30^**^	−.37^**^	−.47^**^	−.60^**^	.73**	.62^**^	.61^**^	.22^**^	.54^**^	.63^**^	–													3.93(3.02)
15. RQ Secure	−.20^**^	−.28^**^	−.13^*^	−.12	.21^**^	.07	.32^**^	−.34**	−.28^**^	−.51^**^	.01	−.14^*^	−.26^**^	−.38^**^	–												4.00(1.85)
16. RQ Fearful	.26^**^	.37^**^	.17^**^	.14^*^	−.18^**^	−.22^**^	−.35^**^	.51**	.42^**^	.58^**^	.13^*^	.24^**^	.44^**^	.48^**^	−.58^**^	–											4.04(1.89)
17. RQ Pre-occupied	.21^**^	.28^**^	.12^*^	.15^*^	−.20^**^	−.29^**^	−.29^**^	.34**	.39^**^	.18^**^	.16^**^	.22^**^	.28^**^	.37^**^	−.10	.16^**^	–										3.31(1.77)
18. RQ Dismissing	.07	.07	.07	.06	.05	−.02	.02	−.01	−.15^*^	.26^**^	−.02	−.13^*^	.00	.01	−.19^**^	.20^**^	−.13^*^	–									4.08(1.75)
19. PRFQ Non-mentalizing	.65^**^	.53^**^	.63^**^	.55^**^	−.47^**^	−.60^**^	−.42^**^	.47**	.41^**^	.45^**^	.23^**^	.25^**^	.37^**^	.39^**^	−.09	.15^*^	.28^**^	.03	–								2.12(.89)
20. PRFQ Certainty	−.29^**^	−.22^**^	−.30^**^	−.24^**^	.48^**^	.29^**^	.20^**^	−.16**	−.15^*^	−.10	−.07	−.15^*^	−.14^*^	−.18^**^	.14^*^	−.06	−.15^*^	.12^*^	−.20^**^	–							3.69(1.01)
21. PRFQ Interest/curiosity	−.19^**^	−.13^*^	−.24^**^	−.12^*^	.24^**^	.14^*^	.08	−.09	−.01	−.14^*^	−.04	−.07	−.08	−.09	.12	.04	.06	−.07	−.26^**^	.21^**^	–						5.76(.75)
22. CTQ-SF Emotional abuse	.21^**^	.23^**^	.18^**^	.15^**^	−.11	−.12^*^	−.18^**^	.34**	.27^**^	.37^**^	.05	.24^**^	.29^**^	.40^**^	−.27^**^	.34^**^	.12^*^	.06	.13^*^	−.08	.03	–					11.96(6.20)
23. CTQ-SF Physical abuse	.08	.11	.08	.02	.01	−.01	−.06	.16**	.10	.20^**^	−.01	.13^*^	.14^*^	.23^**^	−.15^*^	.17^**^	.05	.14^*^	.06	−.02	−.03	.68^**^	–				8.96(5.18)
24. CTQ-SF Sexual abuse	.10	.15^*^	.07	.06	.01	−.05	.01	.10	.05	.12^*^	−.04	.06	.13^*^	.30^**^	−.15^*^	.13^*^	−.04	.06	.09	−.11	−.12^*^	.35^**^	.33^**^	–			8.08(5.64)
25. CTQ-SF Emotional neglect	.26^**^	.20^**^	.23^**^	.25^**^	−.14^*^	−.13^*^	−.18^**^	.29**	.29^**^	.32^**^	.02	.12^*^	.24^**^	.34^**^	−.28^**^	.32^**^	.17^**^	.07	.19^**^	−.05	−.03	.77^**^	.50^**^	.30^**^	–		14.17(3.96)
26. CTQ-SF Physical neglect	.17^**^	.14^*^	.17^**^	.13^*^	−.06	−.06	−.12^*^	.23**	.22^**^	.27^**^	−.02	.09	.20^**^	.29^**^	−.28^**^	.24^**^	.12^*^	.05	.14^*^	−.06	−.07	.63^**^	.59^**^	.35^**^	.69^**^	–	7.95(3.73)

*Notes*. *p < .05, **p < .01. *PSI-4-SF* Parenting Stress Index 4th Edition – Short Form, *PSOC* Parenting Sense of Competency Scale, *PID-5-BF* Personality Inventory for DSM-5 – Brief Form, *MHI-5* Mental Health Inventory, *CTQ-SF* Childhood Trauma Questionnaire – Short Form, *RQ* Relationships Questionnaire, *PRFQ* Parental Reflective Functioning Questionnaire

Multiple linear regression equations modelled the impact on parenting stress and competence variables from several independent variables. Dependent variables included parenting stress (i.e. parenting stress, parenting distress, difficult child, and difficult relationship) and parenting competence (i.e. efficacy and satisfaction). Whilst independent variables included parental mental health (i.e. psychology wellbeing, BPD features and personality traits), trauma history (i.e. emotional neglect, physical neglect, emotional abuse, physical abuse, sexual abuse), attachment style (i.e. secure, pre-occupied, dismissing, fearful) and reflective capacity (i.e. non-mentalizing, certainty about mental states, interest/curiosity in mental states). Independent variables were entered simultaneously. Personality disorder severity was not modelled due to its high correlation with personality disorder trait domain variables, indicating multi-collinearity between the variables. Findings are presented in Tables 4 and 5.

**Table 4:** Multivariate linear regressions modelling parenting stress by mental health variables (*N =* 284)

	Parenting stress domains (PSI-4-SF)															
	Parenting stress				Parenting distress				Difficult child				Difficult relationship			
	*b*	*SE*	*β*	*t*	*b*	*SE*	*β*	*t*	*b*	*SE*	*β*	*t*	*b*	*SE*	*β*	*t*
	*F*(19,283) = 19.76***, *R*^*2*^ = .59				*F*(19,283) = 21.39***, *R*^*2*^ = .61				*F*(19,283) = 9.41***, *R*^*2*^ = .40				*F*(19,283) = 13.29***, *R*^*2*^ = .49			
PID-5-BF Personality traits																
Negative affect	.95	.46	.13	2.08*	.23	.17	.09	1.37	.35	.20	.13	1.70	.37	.20	.13	1.87
Detachment	1.22	.54	.15	2.24*	.70	.20	.23	3.48**	.08	.24	.03	.31	.44	.24	.14	1.85
Antagonism	−.28	.55	−.02	−.51	−.38	.21	−.08	−1.84	−.04	.25	−.01	−.14	.13	.24	.03	.55
Disinhibition	−.18	.47	−.02	−.38	.01	.18	.00	.08	−.09	.21	−.03	−.41	−.11	.21	−.03	−.52
Psychoticism	−.14	.45	−.02	−.32	.03	.17	.01	.17	−.07	.20	−.02	−.33	−.10	.20	−.04	−.54
MSI-BPD BPD Features	−.27	.59	−.03	−.45	.08	.22	.03	.38	−.12	.26	−.04	−.45	−.23	.26	−.07	−.90
MHI-5 Psychological wellbeing	−1.32	.31	−.26	−4.33***	−.77	.11	−.39	−6.74***	−.37	.14	−.20	−2.74**	−.18	.13	−.09	−1.38
CTQ-SF Trauma history																
Emotional abuse	.10	.33	.02	.314	.08	.12	.05	.68	−.01	.15	−.01	−.07	.03	.14	.02	.22
Physical abuse	−.31	.29	−.06	−1.06	−.02	.11	−.01	−.22	−.21	.13	−.11	−1.58	−.08	.13	−.04	−.63
Sexual abuse	.16	.21	.04	.76	.19	.08	.11	2.42**	.01	.10	.00	.07	−.04	.09	−.02	−.39
Emotional neglect	.69	.46	.11	1.49	−.11	.17	−.04	−.64	.54	.21	.22	2.64**	.25	.20	.10	1.26
Physical neglect	−.21	.42	−.03	−.49	−.17	.16	−.07	−1.10	−.09	.19	−.04	−.47	.06	.18	.02	.31
RQ Attachment style																
Secure	.84	.75	.06	1.13	.35	.28	.07	1.24	.19	.33	.04	.57	.31	.33	.06	.95
Fearful	−.15	.79	−.01	−.19	.23	.29	.04	.78	−.29	.35	−.06	−.84	−.09	.34	−.02	−.25
Pre-occupied	−.77	.67	−.05	−1.15	.28	.25	.05	1.11	−.43	.30	−.08	− 1.45	−.61	.29	−.11	−2.09
Dismissing	1.04	.68	.07	1.53	.25	.25	.04	.99	.49	.30	.09	1.61	.30	.30	.05	1.02
PFRQ Reflective capacity																
Non-mentalizing	12.18	1.48	.42	8.24***	2.22	.55	.20	4.04***	4.46	.66	.41	6.77***	5.50	.65	.48	8.53***
Certainty about mental states	−3.88	1.09	−.15	−3.55***	−.63	.41	−.06	−1.54	−1.4	.49	−.15	−2.87**	−1.85	.48	−.18	−3.88***
Interest/curiosity in mental states	−.19	1.51	−.01	−.13	−.02	.56	−.00	−.04	.51	.68	.04	.76	−.68	.66	−.05	−1.03

*Notes*. *p < .05, **p < .01, ***p < .001. *b* unstandardized regression coefficient, *SE* standard error of the mean, *β* standardised regression coefficient, *R*^*2*^ amount of variance accounted for in the dependent variable by each model. PSI-4-SF, Parenting Stress Index 4th Edition – Short Form, *PID-5-BD* Personality Inventory for DSM-5 – Brief Form, *MHI-5* Mental Health Inventory, *CTQ-SF* Childhood Trauma Questionnaire – Short Form, *RQ* Relationships Questionnaire, *PRFQ* Parental Reflective Functioning Questionnaire

**Table 5:** Multivariate linear regressions modelling parenting competence by mental health variables (*N =* 284)

	Parenting competency domains (PSOC)							
	Parenting satisfaction				Parenting efficacy			
	*b*	*SE*	*β*	*t*	*b*	*SE*	*β*	*t*
	*F*(19,283) = 14.15***, *R*^*2*^ = .51				*F*(19,283) = 19.36***, *R*^*2*^ = .58			
PID-5-BF Personality traits								
Negative affect	−.05	.12	−.03	−.44	−.33	.13	−.17	−2.59*
Detachment	−.10	.14	−.05	−.70	−.27	.15	−.12	−1.75
Antagonism	.05	.15	.02	.33	.25	.16	.07	1.62
Disinhibition	−.36	.13	−.17	−2.86**	−.31	.13	−.13	−2.35*
Psychoticism	.20	.12	.11	1.72	.08	.13	.04	.60
MSI-BPD BPD features	.03	.16	.01	.18	.00	.17	.00	.00
MHI-5 Psychological wellbeing	.36	.08	.29	4.42***	.42	.09	.29	4.82***
CTQ-SF Trauma history								
Emotional abuse	.00	.09	.00	.01	.00	.09	.00	.01
Physical abuse	.08	.08	.07	1.08	.09	.08	.06	1.06
Sexual abuse	.07	.06	.07	1.30	−.05	.06	−.04	−.78
Emotional neglect	−.15	.12	−.09	−1.21	−.03	.13	−.02	−.24
Physical neglect	.07	.11	.04	.64	.11	.12	.06	.90
RQ Attachment style								
Secure	.08	.20	.02	.38	−.80	.21	−.20	−3.81***
Fearful	.02	.21	.01	.09	−.08	.22	−.02	−.34
Pre-occupied	.10	.18	.03	.58	−.14	.19	−.03	−.73
Dismissing	−.01	.18	−.00	−.06	−.39	.19	−.10	−2.07*
PFRQ Reflective capacity								
Non-mentalizing	−1.66	.39	−.23	−4.22***	−2.71	.42	−.33	−6.52***
Certainty about mental states	2.21	.29	.36	7.61***	1.13	.31	.16	3.69***
Interest/curiosity in mental states	.60	.40	.07	1.49	−.02	.43	−.00	−.05

*Notes*. *p < .05, **p < .01, ***p < .001. *b* = unstandardized regression coefficient, *SE* standard error of the mean, *β* standardised regression coefficient, *R*^*2*^ amount of variance accounted for in the dependent variable by each model. *PSOC* Parenting Sense of Competency Scale, *PID-5-BF* Personality Inventory for DSM-5 – Brief Form, *MHI-5* Mental Health Inventory, *CTQ-SF* Childhood Trauma Questionnaire – Short Form, *RQ* Relationships Questionnaire, *PRFQ* Parental Reflective Functioning Questionnaire

[In the stress domain, parenting stress was partially explained by greater negative affect and detachment, non-mentalizing, and lesser certainty about mental states and psychological wellbeing, with the model explaining 59% of the variance in stress scores*, F*(19,283) = 19.76, *p* < .001. Parenting distress was partially explained by greater detachment, recalled sexual abuse, non-mentalizing, and lesser psychological wellbeing, with the model explaining 61% of the variance in distress scores, *F*(19,283) = 21.39, *p* < .001. Perceptions of parenting a difficult child was partially explained by greater history of emotional neglect, non-mentalizing, and lesser certainty about mental states and psychological wellbeing, with the final model explaining 40% of the variance in difficult child scores, *F*(19,283) = 9.41, *p* < .001. Perceptions of having a difficult parent-child relationship was partially explained by greater non-mentalizing mode, and lesser certainty about mental states, with the final model explaining 49% of the variance in difficult relationship scores, *F*(19,283) = 13.29, *p* < .001.

In the competence domain, parenting satisfaction was partially explained by greater general psychological wellbeing and certainty about mental states, and lesser non-mentalizing and disinhibition, with the final model explaining 51% of the variance in satisfaction scores, *F*(19,283) = 14.15, *p* < .001. Parenting efficacy was partially explained by greater general psychological wellbeing, secure attachment relationships, and certainty about mental states, and lesser negative affectivity and disinhibition, dismissing-avoidant attachment style, and non-mentalizing, with the final model explaining 58% of the variance in efficacy scores, *F*(19,283) = 19.36, *p* < .001.

## Discussion

The current study compared parents high and low in BPD features on a range of parenting and mental health variables, including personality traits, general psychological wellbeing, trauma history, attachment and reflective capacity. We also examined how parenting stress and competence are associated with personality traits, psychological wellbeing, trauma history, attachment and reflective capacity.

In support of our first hypothesis and in line with previous research [17], individuals high in BPD features (who met caseness for BPD) were found to report significantly greater parenting stress, distress, difficult child and difficult parent-child relationships compared to those with low BPD features. These individuals also reported significantly lower parenting satisfaction and efficacy compared to those lower in BPD features. The findings reported here contribute to a growing body of research exploring the impact of personality disorder and specifically BPD on an individual’s sense of self and role as a parent [13].

In support of our second hypotheses, individuals high in BPD features were also found to endorse greater additional challenges compared to those lower in BPD features. Individuals high in BPD features were found to report significantly greater personality disorder severity using measures from the alternate trait model for DSM-5 [2, 44]. These individuals were also more likely to endorse the personality trait domains of negative affect, detachment, antagonism, disinhibition, and psychoticism. In the current study, presence of BPD features (indicated by number of features endorsed by individuals on the MSI-BPD) was significantly positively correlated with personality disorder severity and personality trait domains (indicated by the individual’s total score and trait scores on the PID-5-BF), supporting the use of dimensional measures based on the alternate model of personality disorder in studies of this type. Individuals with high BPD features were also found to report significantly lower psychological wellbeing on a measure that is frequently used to screen for anxiety and depression [47], a finding in line with the known comorbidity of BPD and general measures of psychopathology [57].

Additionally, individuals with high BPD features also recalled significantly higher emotional abuse, physical abuse, sexual abuse, emotional neglect and physical neglect in their childhood compared those with low BPD features. These findings support the current study’s hypotheses and reinforce an extensive body of research examining the association between early childhood maltreatment and BPD in adolescence and adulthood [13]. Individuals with high BPD features were also more likely to endorse a fearful and preoccupied attachment style and less likely to endorse a secure attachment style than those with low BPD features, a finding concordant with previous studies [24]. However, dismissing-avoidant attachment did not significantly differ between the groups. This discrepancy may be the result of this sample being a group of parents where the majority were married (58.45%) or living with their partner (17.61%), and thus involved in at least one other attachment relationship. This non-significant finding could also reflect that like secure attachment, a dismissing-avoidant style may also be protective [54].

In the current study, individuals with high BPD features also reported a poorer reflective capacity (indicated by increased non-mentalizing and being ‘overly certain’ about mental states) than those with low BPD features. These findings are consistent with a body of research looking at the relationship between BPD and a parent’s ability to hold their child’s mind in mind [34, 35, 58, 59]. However, an individual’s interest and curiosity in their child’s mental states did not significantly differ between the groups, and both low and high BPD groups endorsed a high level of curiosity and interest in the mental states of their children. Given the self-report nature of this questionnaire, this non-significant finding could be related to participants’ reluctance to report a lower interest or curiosity in the mental states of their child due to factors such as social desirability. It is also possible that this interest and curiosity in a child’s mental state does not differentiate those high and low in BPD features.

In partial support of our third hypothesis, the number (or severity) of BPD features did not improve the regression model’s attempt to explain parenting stress or competence, despite BPD features being significantly related in simple correlations. This finding is in contrast to previous research [17], which found that mothers with BPD reported being less satisfied, less competent and more distressed than community controls. Yet some personality trait domains, namely negative affectivity, detachment and disinhibition (which are also implicated in the DSM-5 criteria for BPD) [2], were associated with parenting stress, distress, difficult parent-child relationship, satisfaction and efficacy in the current study. As such, the specific personality traits domains in the alternative or dimensional models of personality disorder (measured using the PID-5-BF) may be more sensitive than the number of BPD characteristics endorsed (measured using the MSI-BPD) for this population. Parenting stress (i.e. parenting stress, distress, and perceptions of having a difficult child) and parenting competence variables (i.e. satisfaction and efficacy) were found to be associated with lower general psychological wellbeing. This inverse relationship between current psychological wellbeing and parenting stress and competence variables is supported by previous research exploring the relationship between mental wellbeing and stress and satisfaction in parents [41, 60].

In further support of our third hypothesis, parenting distress and perceptions of having a difficult child were associated with parent’s recalled traumatic early experiences, specifically sexual abuse and emotional neglect in the family environment. There was a positive relationship between sexual abuse and parenting stress, and emotional neglect and difficult child, in that higher sexual abuse and emotional neglect recalled by parents was found to increase parenting stress and perceptions of parenting a difficult child in the present. This significant finding aligns with the literature exploring the possible impact of childhood trauma on parenting capacity in adulthood [61]. Having a lived experience of trauma may amplify the challenges parents already face. A parent who recalls history of early maladaptive parenting may experience post-traumatic symptoms triggered by the presence or demands of their child [62], resulting in increased distress and increased perception of parenting a ‘difficult child’.

In partial support of our third hypothesis, we found that greater attachment security was associated with greater parenting efficacy. This finding is in support of previous research which suggests that secure working models of attachment promote and sustain effective caregiving and parenting self-esteem [63]. However, parent’s attachment style did not add to the regression model for either parenting stress variables or parenting satisfaction. This may be related to adult attachment being measured dimensionally by style, rather than categorically (e.g. secure vs. insecure), in the present study.

In further support of our third hypothesis, parental reflective capacity or their ability to ‘hold the child’s mind in mind’ was found to be associated with parenting stress and competence variables. According to Luyten et al. [56], the ‘non-mentalizing’ subscale of the PRFQ captures a non-mentalizing stance, malevolent attributions and an inability to enter the subjective world of the child. In the current study we found that poorer reflective capacity (indicated by increased non-mentalizing) was related to greater parenting stress, distress, perception of parenting a difficult child, and having a difficult parent-child relationship. Greater reflective capacity (indicated by decreased non-mentalizing) was associated with greater parenting satisfaction and efficacy. Notably, non-mentalizing had the strongest association with parenting stress, perceptions of having a difficult child, difficult parent-child relationship and parenting efficacy. It therefore appears that a lesser ability (or inability) to enter the subjective world of the child increases parenting stress, and conversely that a greater ability to enter the subjective world of the child has a positive impact on parenting satisfaction and efficacy. Difficulty holding other’s minds in mind [64] and the tendency to create malevolent attributions to neutral stimuli [65, 66] have previously been implicated in research exploring the impact of personality disorder, particularly BPD, on these higher order cognitive processes. Parents who have deficits in reflective capacity are also at increased risk of having offspring who exhibit disorganised attachment in childhood, and BPD in adulthood [67]. Moreover, lower certainty about mental states (indicating the parent was ‘overly certain’) was found to be associated with increased parenting stress, perceptions of parenting a difficult child and difficult parent-child relationship. Whilst increased certainty (indicating the parent was ‘lacking in certainty’) was found to be associated with greater parenting satisfaction and efficacy. Notably, certainty about mental states had the strongest association with parenting satisfaction. In the PRFQ [56], the ‘certainty about mental states’ subscale reflects a parent’s ability to recognise the opacity of mental states. Lower scores on this scale reflect a tendency for parents to be overly certain about mental states of their child (i.e. intrusive mentalizing or hyper-mentalizing) and higher scores reflecting a lack of certainty about their child’s mental states. Our finding that lacking in certainty about a child’s mental states increases parenting satisfaction and efficacy may be indicative of a parent having a more open, flexible and reflective understanding of the subjective world of their child, creating a parent-child relationship that is experienced as satisfying or fulfilling for the parent. We believe that that such a relationship would also have a positive impact on the child, and may help negate the putative transmission of mental health difficulties from parents to their children.

There are however a number of limitations to the current study that must be considered when interpreting these results. First, this research was reliant on self-report measures. Therefore, individuals who endorsed high BPD features and meet caseness may not be generalisable to a clinical BPD population. Although we measured general psychological wellbeing, we did not control for the high-comorbidity in diagnoses with BPD (e.g. mood disorders, substance use disorders and other personality disorders). We also did not control for the number, age or gender of children, which may have had an impact of individual’s self-reported parenting stress and competency. Moreover, we relied on two relatively novel self-report measures to assess personality pathology (PID-5-BF) [2] and reflective capacity (PRFQ) [56] and as such, further research confirming the reliability of these measures is needed. Additionally, we used a cross-sectional design for this research and therefore we can only infer correlational relationships and not causal relationships between parenting stress and competence and personality and mental health, trauma history, attachment and reflective capacity. Future longitudinal research is therefore needed to test for causal relationships between parenting stress and competence and the potential underlying mechanism we have identified in a clinical BPD sample, a psychiatric control and a general population control group.

## Conclusion

Individuals who met caseness for BPD experienced challenges in their parenting but also associated difficulties in their general psychological wellbeing, attachment and reflective capacity. These individuals were also more likely to report a history trauma in their family environment, including abuse and neglect. For the entire sample, poorer reflective capacity (indicated by increased non-mentalizing and being ‘overly certain’ about mental states), psychological wellbeing and specific personality traits (namely negative affect, disinhibition and detachment) had the strongest association with parenting stress and competence domains. These findings suggest that parents who were able to imaginatively enter the subjective inner world of the child had less stress and greater parenting satisfaction and efficacy. In combination, genetic vulnerability to BPD and negative early experiences with parents and caregivers are considered to put a child at increased risk of developing BPD or experiencing its related features in adulthood [11, 12]. However, parenting practices have the ability to be modified and thus offer an important context for the development of interventions broadly aiming to improve the parent-child relationship. In order to decrease parenting stress and increase parenting competence it appears that we need to enhance a parent’s capacity to hold their child’s mind in mind and their attachment relationships, whilst also addressing their general psychological wellbeing and specific personality traits that place them at risk. In the context of clinical practice, a number of key strategies, including addressing and reducing BPD symptoms, increasing parental reflective capacity and strengthening parent-child attachment relationships, may be implemented to reduce parenting stress and increase parenting competence in at risk parents. We anticipate that this would have a positive impact on not only parenting capacity, but also the parent-child relationship and reducing the risk for the potential intergenerational transmission of BPD.

## Data Availability

The datasets used and/or analysed during the current study are available from the corresponding author on reasonable request.

## Abbreviations

BPD: Borderline Personality Disorder
CTQ-SF: Childhood Trauma Questionnaire – Short Form
DSM-5: Diagnostic and Statistical Manual for Mental Disorders – 5th Edition
MHI-5: Mental Health Inventory – 5 item
PID-5-BF: Personality Inventory for DSM-5 – Brief Form
PRFQ: Parental Reflective Functioning Questionnaire
PS-4-SF: Parenting Stress Index 4th Edition – Short Form
PSOC: Parenting Sense of Competency Scale
RQ: Relationships Questionnaire

## References

[1] World Health Organization (WHO). International Statistical Classification of Diseases and Related Health Problems (11th edition). Geneva: Author; 2019. https://icd.who.int/browse11/l-m/en. Accessed 12 Feb 2020.

[2] American Psychiatric Association (2013). Diagnostic and statistical manual of mental disorders.

[3] Winsper C, Bilgin A, Thompson A, Marwaha S, Chanen AM, Singh SP, Wang A, Furtado V (2019). The prevalence of personality disorders in the community: a global systematic review and meta-analysis. Br J Psychiatry.

[4] Grenyer BF, Ng FY, Townsend ML, Rao S (2017). Personality disorder: a mental health priority area. Aust N Z J Psychiatry.

[5] National Health and Medical Research Council (2012). Clinical practice for the management of borderline personality disorder.

[6] Torgersen S, Widiger TA (2012). Epidemiology. The Oxford handbook of personality disorders.

[7] Grant BF, Chou SP, Goldstein RB (2008). Prevalence, correlates, disability, and comorbidity of DSM-IV borderline personality disorder: results from the wave 2 National Epidemiologic Survey on alcohol and related conditions. J Clin Psychiatry.

[8] Skodol AE, Bender DS (2003). Why are women diagnosed borderline more than men?. Psychiatry Q.

[9] Grenyer BFS (2014). An integrative relational step-down model of care: the project air strategy for personality disorders. ACPARIAN..

[10] Ronningstam EF, Keng SL, Ridolfi ME, Arbabi M, Grenyer BFS (2018). Cultural aspects in symptomatology, assessment, and treatment of personality disorders. Current Psychiatry Reports.

[11] Leichsenring F, Leibing E, Kruse J, New AS, Leweke F (2011). Borderline personality disorder. Lancet..

[12] Winsper C (2018). The aetiology of borderline personality disorder (BPD): contemporary theories and putative mechanisms. Curr Opin Psychol.

[13] Steele KR, Townsend ML, Grenyer BFS (2019). Parenting and personality disorder: an overview and meta-synthesis of systematic reviews. PLoS One.

[14] Barnow S, Spitzer C, Grabe HJ, Kessler C, Freyberger HJ (2006). Individual characteristics, familial experience, and psychopathology in children of mothers with borderline personality disorder. J Am Acad Child Adolesc Psychiatry.

[15] Crandell LE, Patrick MPH, Hobson RP (2003). 'Still-face' interactions between mothers with borderline personality disorder and their 2-month-old infants. Br J Psychiatry.

[16] Hobson RP, Patrick MPH, Hobson JA, Crandell L, Bronfman E, Lyons-Ruth K (2009). How mothers with borderline personality disorder relate to their year-old infants. Br J Psychiatry.

[17] Newman LK, Stevenson CS, Bergman LR, Boyce P (2007). Borderline personality disorder, mother-infant interaction and parenting perceptions: preliminary findings. Aust N Z J Psychiatry.

[18] Blankley G, Galbally M, Snellen M, Power J, Lewis AJ (2015). Borderline personality disorder in the perinatal period: early infant and maternal outcomes. Australasian Psychiatry.

[19] Hobson RP, Patrick M, Crandell L, García-Pérez R, Lee A (2005). Personal relatedness and attachment in infants of mothers with borderline personality disorder. Dev Psychopathol.

[20] MacFie J, Swan SA (2009). Representations of the caregiver-child relationship and of the self, and emotion regulation in the narratives of young children whose mothers have borderline personality disorder. Dev Psychopathol.

[21] Zanarini MC, Yong L, Frankenburg FR, Hennen J, Reich DB, Marino MF (2002). Severity of reported childhood sexual abuse and its relationship to severity of borderline psychopathology and psychosocial impairment among borderline inpatients. J Nerv Ment Dis.

[22] Laporte L, Paris J, Zelkowitz P (2018). Estimating the prevalence of borderline personality disorder in mothers involved in youth protection services. Personal Ment Health.

[23] Fonagy P, Target M, Gergely G (2000). Attachment and borderline personality disorder. A theory and some evidence. Psychiatr Clin N Am.

[24] Agrawal HR, Gunderson J, Holmes BM, Lyons-Ruth K (2004). Attachment studies with borderline patients: a review. Harvard Review Psychiatry.

[25] Bateman A, Fonagy P (2004). Mentalisation based treatment of borderline personality disorder. J Personal Disord.

[26] Levy KN, Beeney JE, Temes CM (2011). Attachment and its vicissitudes in borderline personality disorder. Curr Psychiatry Reports.

[27] Katznelson H (2014). Reflective functioning: a review. Clin Psychol Reviw.

[28] Goodman M, Siever LJ (2011). Hypermentalization in adolescents with borderline personality traits: extending the conceptual framework to younger ages. J Am Acad Child Adolesc Psychiatry.

[29] Goodman G (2013). Is mentalization a common process factor in transference-focused psychotherapy and dialectical behavior therapy sessions?. J Psychother Integr.

[30] Diamond D, Levy KN, Clarkin JF, Fischer-Kern M, Cain NM, Doering S (2014). Attachment and mentalization in female patients with comorbid narcissistic and borderline personality disorder. Personal Disord Theory Res Treat.

[31] Fonagy P, Gergely G, Jurist EL, Target M (2002). Affect regulation, mentalization, and the development of the self.

[32] Allen JG, Fonagy P, Bateman A (2008). Mentalizing in clinical practice.

[33] Luyten P, Fonagy P, Lowyck B, Vermote R, Bateman A, Fonagy P (2012). Assessment of mentalization. Handbook of mentalizing in mental health practice.

[34] Slade A (2005). Parental reflective functioning: an introduction. Attachment Human Development..

[35] Slade A, Grienenberger J, Bernbach E, Levy D, Locker A (2005). Maternal reflective functioning, attachment, and the transmission gap: a preliminary study. Attachment Human Development.

[36] Ainsworth MDS, Wittig BA, Foss BM (1969). Attachment and exploratory behavior of one-year-olds in a strange situation. Determinants of infant behaviour.

[37] Fonagy E, Steele M, Steele H, Leigh T, Kennedy R, Mattoon G, Target M, Goldberg S, Muir R, Kerr K (1995). The predictive validity of Mary Main’s adult attachment interview: a psychoanalytic and developmental perspective on the transgenerational transmission of attachment and borderline states. Attachment theory: social, developmental and clinical perspectives.

[38] Marcoux AA, Bernier A, Seguin JR, Boike-Armerding J, Lyons-Ruth K (2017). How do mothers with borderline personality disorder mentalize when interacting with their infants?. Personal Ment Health.

[39] Pederson DR, Gleason KE, Moran G, Bento S (1998). Maternal attachment representations, maternal sensitivity, and the infant-mother attachment relationship. Dev Psychol.

[40] Zanarini MC, Vujanovic A, Parachini EA, Boulanger JL, Frankenburg FR, Hennen J (2003). A screening measure for BPD: the McLean screening instrument for borderline personality disorder. J Personal Disord.

[41] Abidin RR (2012). Parenting stress index, fourth edition (PSI-4).

[42] Gibaud-Wattston I, Wandersman LP (1978). Development and utility of the parenting sense of competence scale.

[43] Johnston C, Mash EJ (1989). A measure of parenting satisfaction and efficacy. J Clin Child Psychol.

[44] Anderson JL, Sellbom M, Salekin RT (2016). Utility of the personality inventory for DSM-5–brief form (PID-5-BF) in the measurement of maladaptive personality and psychopathology. Assessment..

[45] Leung SW, Leung F (2009). Construct validity and prevalence rate of borderline personality disorder among Chinese adolescents. J Personal Disord.

[46] Patel AB, Sharp C, Fonagy P (2011). Criterion validity of the MSI-BPD in a community sample of women. J Psychopathol Behav Assess.

[47] Berwick D, Murphy J, Goldman P, Ware J, Barsky A, Weinstein M (1991). Performance of a five-item mental health screening test. Med Care.

[48] Rivera-Riquelme M, Piqueras JA, Cuijpers P (2019). The revised mental health Inventory-5 (MHI-5) as an ultra-brief screening measure of bidimensional mental health in children and adolescents. Psychiatry Res.

[49] Bernstein DP, Fink L (1998). Childhood trauma questionnaire: a retrospective self-report manual.

[50] Bartholomew K, Horowitz LM (1991). Attachment styles among young adults: a test of a four-category model. J Pers Soc Psychol.

[51] Bowlby J (1988). A secure base: parent-child attachment and healthy human development.

[52] Bartholomew K, Shaver PR, Simpson JA, Rholes WS (1998). Methods of assessing adult attachment: do they converge?. Attachment theory and close relationships.

[53] Reis S, Grenyer BFS. Pathways to anaclitic and introjective depression. Psychology and Psychotherapy: Theory, Research and Practice. 2002;75(4):445–59. 10.1348/147608302321151934 12626134

[54] Reis S, Grenyer BFS (2004). Fearful attachment, working Alliance and treatment response for individuals with major depression. Clin Psychol Psychotherapy.

[55] Choi-Kain LW, Fitzmaurice GM, Zanarini MC (2009). The relationship between self-reported attachment styles, interpersonal dysfunction, and borderline personality disorder. J Nerv Ment Dis.

[56] Luyten P, Mayes LC, Nijssens L, Fonagy P (2017). The parental reflective functioning questionnaire: development and preliminary validation. PLoS One.

[57] Skodol AE, Gunderson JG, Pfohl B, Widiger TA, Livesley WJ, Siever LJ (2002). The borderline diagnosis I: psychopathology, comorbidity, and personality structure. Biol Psychiatry.

[58] Grienenberger JF, Kelly K, Slade A (2005). Maternal reflective functioning, mother-infant affective communication, and infant attachment: exploring the link between mental states and observed caregiving behavior in the intergenerational transmission of attachment. Attach Hum Dev.

[59] Sharp C, Fonagy P (2008). The parent's capacity to treat the child as a psychological agent: constructs, measures and implications for developmental psychopathology. Soc Dev.

[60] Albanese AM, Russo GR, Geller PA. The role of parental self-efficacy in parent and child well-being: A systematic review of associated outcomes. Child: Care, Health and Development. 2019;45(3):333–63. 10.1111/cch.12661 30870584

[61] Chamberlain et al. Parenting after a history of childhood maltreatment: A scoping review and map of evidence in the perinatal period. PLOS ONE. 2019;14(3): e0213460. 10.1371/journal.pone.0213460 PMC6415835 30865679

[62] Newman L, Stevenson C (2005). Parenting and Borderline Personality Disorder: Ghosts in the Nursery. Clin Child Psychol Psychiatry.

[63] Mikulincer M, Shaver PR (2007). Attachment in adulthood: structure, dynamics, and change.

[64] Fonagy P, Bateman AW (2006). Mechanisms of change in mentalization-based treatment of BPD. J Clin Psychol.

[65] Roepke S, Vater A, Preißler S, Heekeren HR, Dziobek I (2013). Social cognition in borderline personality disorder. Front Neurosci.

[66] Winter D, Herbert C, Koplin K, Schmahl C, Bohus M, Lis S (2015). Negative evaluation Bias for positive self-referential information in borderline personality disorder. PLoS One.

[67] Nijssens L, Luyten P, Bales DL, Midgley N, Vrouva I (2012). Mentalization-based treatment for parents (MBT-P) with borderline personality disorder and their infants. Minding the child: Mentalization-based interventions with children, young people and their families.

